# Supplementary material for: Telomere Length in Pig Sperm Is Related to In Vitro Embryo Development Outcomes
Source: Animals (Basel). 2022 Jan 15;12(2):204. doi: 10.3390/ani12020204 (PMC8773156; doi:10.3390/ani12020204)
Supplement: Supplementary file 1 [file animals-12-00204-s001.zip › animals-1530863-supplementary.pdf]

Table S1. Telomere length obtained for each boar. # indicates boars used for IVF.

Boar	Telomere length (Mean ± SD)
1 #	10.39 ± 5.98
2 #	18.18 ± 3.99
3 #	18.33 ± 6.12
4 #	18.84 ± 6.26
5 #	19.46 ± 5.57
6	20.21 ± 1.66
7 #	20.97 ± 7.57
8	21.41 ± 3.73
9	21.74 ± 2.47
10	21.81 ± 2.29
11	21.94 ± 3.77
12 #	22.04 ± 4.18
13	22.06 ± 2.44
14 #	23.00 ± 6.16
15 #	23.12 ± 7.46
16	23.94 ± 5.96
17	24.26 ± 4.15
18	24.58 ± 4.46
19 #	24.85 ± 5.60
20 #	25.13 ± 4.72
21	25.17 ± 3.50
22 #	29.01 ± 9.49
23 #	29.17 ± 8.17

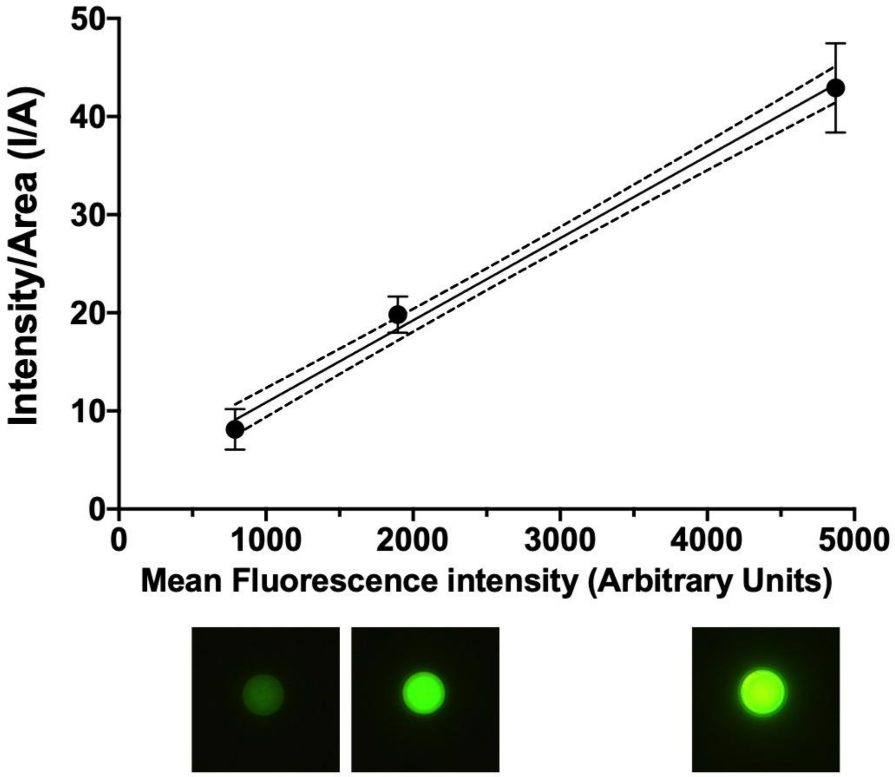

Figure S1. Calibration line performed with fluorescence particles to extrapolate arbitrary fluorescence intensity to fluorochrome number. Images show a representative particle of the three intensities used.
